# Current Understanding of the Pathophysiology of Idiopathic Intracranial Hypertension

**DOI:** 10.1007/s11910-025-01420-y

**Published:** 2025-04-16

**Authors:** Michael Lowe, Gabriele Berman, Priya Sumithran, Susan P. Mollan

**Affiliations:** 1 Department of Neurology, Institute of Neurological Sciences, Queen Elizabeth University Hospital, Glasgow, G51 4 TF UK; 2 Birmingham Neuro-Ophthalmology, University Hospitals Birmingham NHS Foundation Trust, Birmingham, B15 2GW UK; 3 Dept of Surgery, School of Translational Medicine, Monash University, Melbourne, 3004 Australia; 4 Dept of Endocrinology and Diabetes, Alfred Health, Melbourne, 3004 Australia; 5 Metabolism and Systems Research, School of Medical Sciences, University of Birmingham, Birmingham, Edgbaston B15 2 TT UK

**Keywords:** Cerebrospinal fluid, Female sex, Glucocorticoid dysregulation, Intracranial pressure, Obesity, Papilloedema

## Abstract

**Purpose of Review:**

Development of safe targeted therapies for idiopathic intracranial hypertension requires a thorough understanding of recent evidence discovering the pathophysiology of the condition. The aim is to provide a review of studies that inform on the underpinning mechanisms that have been associated with idiopathic intracranial hypertension.

**Recent Findings:**

People living with active idiopathic intracranial hypertension and obesity have been found to have with insulin resistance, hyperleptinaemia, and adverse cardiovascular outcomes. Clinically their adipose tissue is predominantly located in the truncal region and on detailed laboratory analysis the cells are primed for weight gain. There is evidence of androgen excess, altered glucocorticoid regulation and changes in pro-inflammatory cytokines. There are distinct alterations in metabolic pathways found in serum, urine and cerebrospinal fluid, that resolve following disease remission. These findings are associated with raised intracranial pressure and are likely secondary to cerebrospinal fluid hypersecretion.

**Summary:**

Idiopathic intracranial hypertension has a profile of systemic metabolic changes, endocrine dysfunction and cardiovascular risk profile distinct from that associated with obesity alone. These systemic metabolic changes are likely to contribute to dysregulation of cerebrospinal fluid dynamics, primarily hypersecretion but with a possible additional effect of reduced clearance resulting in the core feature of raised intracranial pressure.

## Introduction

**Fig. 1:**
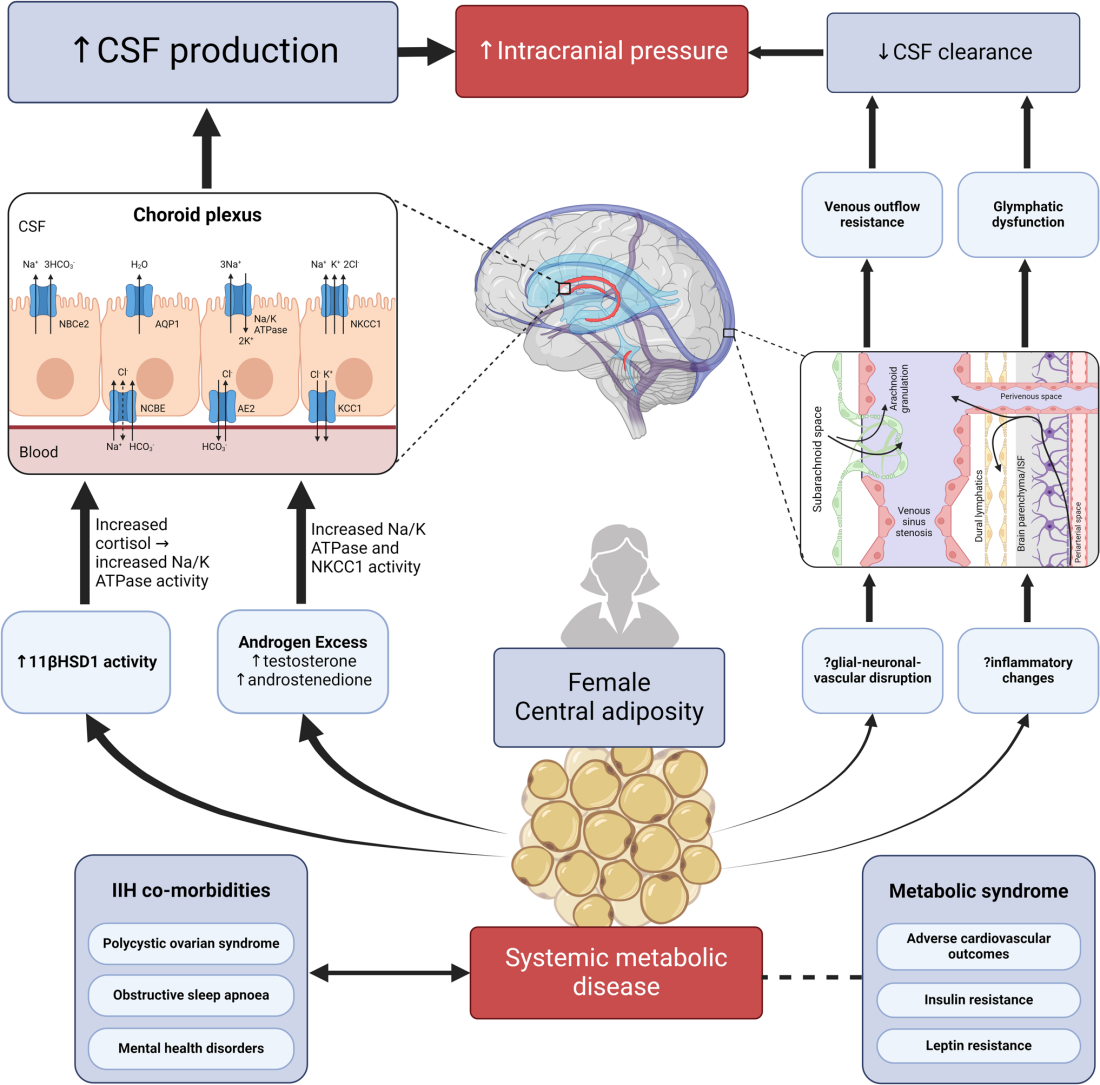
Proposed pathophysiology of IIH. IIH is primarily a systemic metabolic disease associated with central adiposity in females. This is associated with a variety of adverse metabolic features including insulin and leptin resistance and adverse cardiovascular outcomes; as well as co-morbidities including polycystic ovarian syndrome, obstructive sleep apnoea and mental health disorders. Metabolic changes in IIH lead to raised ICP – we propose that increased CSF production at the choroid plexus mediated by androgen excess and changes in corticosteroid activity is the most important driver of this. The left hand side of the diagram outlines these pathways, and inset is a schema of important transporters involved in CSF secretion at the choroid plexus. On the right, there is also reduced CSF clearance due to outflow resistance which may be mediated by inflammatory changes causing glial-neuronal-vascular disruption and glymphatic dysfunction; as well as increased venous outflow resistance and venous sinus stenosis. The inset schema highlights major pathways of CSF clearance via the arachnoid granulations into the venous sinuses, and via parenchymal perivascular spaces of the glymphatic pathway into dural lymphatics and/or venous sinuses

Idiopathic Intracranial Hypertension (IIH) is a challenging condition characterised by raised intracranial pressure (ICP), papilloedema, with risk of permanent visual loss and chronic headaches which reduce quality of life [1, 2]. IIH predominately affects young women, typically in their reproductive years [3, 4]. It is principally associated with obesity, which may add to stigma and create a barrier to management [5, 6] It is acknowledged that there are different spectrums of the disease: the paediatric condition is unlikely to have similar pathophysiology driver as the adolescent and adult disease [7]. It could also be postulated that those that live with IIH but not obesity may have alternative instigating mechanisms that give rise the syndrome of IIH, such as venous sinus stenosis [8, 9]. As research has developed in this condition, there have been new data to implicate that those adults who live with obesity and IIH have a profile of metabolic changes, endocrine dysfunction and cardiovascular risk distinct from that associated with obesity alone [10]. These systemic metabolic changes likely contribute to dysregulation of cerebrospinal fluid (CSF) dynamics, summarised in Fig. 1, and give rise to the signs and symptoms of IIH.

### Adipose Tissue is Predominantly Truncal and Primed for Weight Gain

Whilst IIH is strongly associated with obesity and female sex, the majority of women living with obesity do not develop IIH. The differences in adiposity characteristics between people with both obesity and IIH and those with obesity alone have been explored. Initial reports, based on waist-hip circumference ratio, suggested IIH was associated with greater lower body adiposity compared with generalised obesity [11]. A subsequent study utilised dual-energy X-ray absorptiometry (DEXA) to more accurately characterise adipose distribution in IIH and found, to the contrary, that the distribution was no different to control participants living with obesity who were matched for sex and body mass index (BMI) [12]. The DEXA imaging demonstrated a higher truncal fat:lean mass ratio in the IIH participants compared with controls [12]. In the literature a truncal pattern of adiposity has been associated with other cardiometabolic diseases [13].

An extensive study in 2021 [14] evaluated adipose tissue in subjects with IIH and matched controls with obesity. Subcutaneous and omental biopsies enabled detailed interrogation of adipose tissue. Transcriptional profiling identified gene expression changes in keeping with lipogenesis, despite biopsies having been obtained in the fasting state. Metabolomic alterations also suggested increased capacity for uptake of branch-chain amino acids which could support lipogenesis. Overall these findings suggest adipose tissue in IIH is primed for lipogenesis and weight gain. A possible theory as to how this occurs may be the failure of the adipose organ’s plasticity to cope with the physiologic stimuli of rapid weight gain which induces the striking alterations in the adipose tissue metabolism, structure, and biodistribution.

### IIH is Associated with Insulin Resistance, Hyperleptinaemia, and Adverse Cardiovascular Outcomes

Insulin resistance, with raised fasting insulin levels [14], has been observed in IIH greater than age- and BMI-matched controls. Insulin resistance is a well-established feature of the metabolic syndrome [15], which is associated with dyslipidaemia, type 2 diabetes and adverse cardiovascular outcomes. A large cohort study evaluated cardiovascular outcomes in 2760 patients with IIH compared to 27,125 age- and BMI-matched controls [3]. This demonstrated higher risks of cardiovascular disease (heart failure, ischaemic heart disease and stroke/transient ischaemic attack) with an adjusted hazard ratio [aHR] of 2.10 [95% CI, 1.61–2.74; p < 0.001]. Greater risks of hypertension and type 2 diabetes were also found. People living with IIH also have an increased risk of gestational diabetes [16].

Leptin is a peptide hormone which is secreted from adipose tissue and has an important role in hypothalamic regulation of satiety and energy homeostasis. IIH patients demonstrate hyperleptinaemia [14, 17–19], and adipose tissue in IIH patients has increased leptin secretion compared with BMI- and sex-matched controls [14]. High CSF leptin levels have also been reported in IIH [18, 19], raising the possibility that hypothalamic leptin resistance could be feature of IIH [20]. However, this has not been a consistent finding [14] and its significance is therefore uncertain.

### IIH is Associated with Distinct Alterations in Metabolic Pathways

Metabolomic analyses have been performed in IIH, demonstrating changes in metabolic pathways compared to age- and BMI-matched controls. One study, utilising nuclear magnetic resonance spectroscopy, found lower urine urea, raised serum lactate:pyruvate ratio and changes in CSF ketone body metabolites in IIH participants [21]. Many of these changes normalised at 12-months following a weight loss intervention with bariatric surgery [21, 22].

A further exploration of metabolic changes in IIH utilised ultrahigh-performance liquid chromatography-mass spectrometry to perform an untargeted metabolomic analysis in serum and CSF of patients with IIH and healthy controls [23]. This identified changes in acylpyruvates, with lower CSF level and raised serum levels in IIH. Alterations in multiple lipid and amino acid metabolites were also demonstrated. Correlations with clinical parameters, including visual function, lumbar puncture opening pressure, papilloedema and headache were found with some metabolites. These changes normalised over 12 months following weight loss with bariatric surgery.

Metabolic pathways have also been linked to ICP reduction after weight loss. One study evaluated ICP reduction with three different methods of bariatric surgery: Roux-en-Y gastric bypass (RYGB), gastric banding and sleeve gastrectomy [24]. It found greater reduction in ICP with RYGB compared to sleeve gastrectomy at two weeks, despite similar weight loss. Interrogating changes in metabolic pathways between these groups found greater post-prandial glucagon-like peptide- 1 (GLP- 1) secretion with RYGB compared with sleeve gastrectomy. This, coupled with evidence that GLP- 1 receptors are present in the choroid plexus, suggests that GLP- 1 may modulate ICP. Indeed an early phase randomised control trial evaluating the GLP- 1 receptor agonist, exenatide, found it reduced ICP in active IIH [25]. Greater dynamic changes in circulating lipid metabolites (ceramides, glycerophospholipids and lysoglycerophospholipids) were also seen with RYGB surgery compared to sleeve or banding surgery, correlating with greater ICP reduction.

Together, these studies suggest that unique systemic metabolic changes occur in IIH, correlate with clinical disease parameters, normalise following therapeutic intervention and can determine treatment response independent of weight loss.

### IIH is Associated with Androgen Excess

Adipose tissue has a well-recognised endocrine function [26]. Adipocytes express several enzymes involved in steroidogenesis [27] and have a role in pre-receptor activation and inactivation of androgens, which is tightly regulated [28]. Excess androgen generation has been reported in obesity [29], and adipose tissue is one source of the hyperandrogenism seen in polycystic ovarian syndrome (PCOS) [30, 31]. In addition to this, in women androgens act on adipocytes to promote adipose accumulation, hypertrophy and insulin resistance, compounding the adverse phenotype.

Due to these observations, the role of androgens has been investigated in IIH. Hyperandrogenism has been associated with an earlier age of onset in IIH [32], although no correlation was found with BMI or duration of IIH in this study. PCOS is recognised as a common co-morbidity in IIH [33, 34] and, whilst not associated with visual or headache outcomes, is associated with infertility [34]. Raised serum and CSF testosterone levels have been reported in IIH compared with controls [19, 35].

One study evaluated androgen levels in patients with IIH compared with age-, sex- and BMI-matched controls with either obesity alone or PCOS [35]. It found that patients with IIH had significantly elevated serum testosterone and reduced androstenedione compared with both PCOS and obesity, along with increased systemic activity of the androgen activating enzyme 5α-reductase [35]. Androstenedione is a naturally produced steroid hormone which serves as an intermediate in the biosynthesis of testosterone. CSF androgens were also measured in IIH compared with obese and lean controls and those with obesity, with higher testosterone and androstenedione levels found in IIH, although these did not correlate with BMI or clinical measures of IIH disease activity [35]. A recognized biological phenomenon is that of differential systemic and target tissue-specific hormone concentrations, such as noted here with reduced serum but increased CSF androstenedione. This may provide insights into the pathophysiology of IIH whereby high CSF androstenedione concentrations provide a pool of androgen precursors for activation to testosterone by the choroid plexus.

### IIH is Associated with Altered Glucocorticoid Regulation

Glucocorticoids are regulated systemically by the hypothalamo-pituitary-adrenal axis, but is also regulated at a tissue level by two 11-beta-hydroxysteroid dehydrogenases [11βHSD]. 11βHSD1 converts the inactive cortisone to active cortisol and is expressed widely, particularly in liver, adipose tissue, gonads and brain [36, 37]; 11βHSD2 inactivates cortisol to cortisone and is important in determining mineralocorticoid specificity in tissues [36].

Obesity is associated with changes in 11βHSD1 activity, with impaired activity in liver but increased activity in adipose tissue [38, 39]. In IIH, 11βHSD1 activity has been demonstrated to be elevated, systemically as well in adipose tissue, when compared to matched controls [40] suggesting excess activity above that related to obesity alone. 11βHSD1 activity is reduced following therapeutic weight loss and correlates with reduction in ICP [40, 41].

### IIH is Associated with Changes in Pro-Inflammatory Cytokines

In obesity, systemic inflammatory changes are well recognised [42]. Adipose tissue is a source of cytokines, including tumour necrosis factor alpha [TNFα], interleukin [IL]− 6, IL- 8, IL- 1β and CC-motif chemokine ligand 2 [CCL2]] and others [42, 43]. Production and release of these cytokines is activated by adipocyte hypertrophy and insulin resistance in obesity, as well as accumulation of pro-inflammatory macrophages in adipose tissue [42].

Inflammatory mechanisms have been explored in IIH. Various studies have measured serum and CSF cytokine levels in IIH, although with variable comparator populations. An early study found that CCL2 was elevated in CSF in IIH compared to healthy controls that were not BMI matched [17]. A subsequent study did not replicate this and found lower serum CCL2 in IIH, but the comparator group was heterogenous including multiple sclerosis and other neurological disorders which may have different inflammatory profiles [18]. Another study evaluating serum cytokines in IIH compared to matched controls found no difference in CCL2 levels but raised IL- 1β, IL- 8 and reduced TNFα [44]. Three other studies, however, reported raised serum TNFα in IIH [45–47], with two studies also reporting raised serum IL- 4 and IL- 10 [46, 47]. Interestingly, in one study TNFα negatively correlated with lumbar puncture opening pressure [45]. IL- 6 [48] has also been reported to be elevated in CSF in IIH. A further study found raised CSF IL- 2 and IL- 17, with a relative increase in CSF:serum ratio compared to CSF:serum albumin ratio, suggestive of intrathecal synthesis of these cytokines [49].

Taken together, these studies indicate that IIH is associated with changes in pro-inflammatory cytokine expression in serum and CSF which differ from those seen in various control groups and other neurological conditions. Given the variable findings to date, it will be important to clarify any unique inflammatory cytokine signature in IIH compared to that associated with obesity alone.

## Systemic Changes in IIH Alter CSF Production and Clearance Causing Raised ICP

IIH, therefore, is associated with a unique phenotype of obesity-related metabolic dysregulation. The mechanisms by which these changes lead to raised ICP, have been uncertain (hence the term ‘idiopathic’) but current research now provides valuable insights.

ICP is related to the volume of the three major components of the intracranial space: brain parenchymal tissue; blood and vasculature; and the CSF. Contained within the rigid skull, changes in volume of these components results in a corresponding change in ICP; this is referred to as the Monro-Kellie doctrine [50]. In IIH, as diagnostic criteria exclude causes of increased tissue or blood volume, it is presumed that raised ICP is driven by an excess CSF volume.

### Physiology of CSF Production

CSF is primarily produced in the choroid plexus – a highly vascular tissue residing within all the ventricles in the brain [51]. In adult humans the choroid plexus produces up to 500 mL of CSF per day, with the static volume of CSF (100 - 150 mL) circulating three to four times per day [52]. A relatively small volume of CSF is thought to be produced at extrachoroidal sites [53].

The mechanisms of CSF secretion at the choroid plexus, and movement of water in the brain generally, are interesting and controversial. Water must pass between different compartments: blood, CSF and brain parenchyma; which are regulated by cellular barriers including the blood–brain barrier (BBB) and blood-CSF barrier [54, 55]. Traditionally, it was thought that water moved by simple diffusion driven by osmotic forces across the cell membrane. The discovery of aquaporins, transmembrane proteins, observed a more efficient way to move water across a cell membrane.

Aquaporin- 1 is of particular interest to CSF secretion, being predominantly localised to the luminal membrane of choroid plexus epithelial cells (CPECs) [54]. The Na^+^-K^+^ Adenosine triphosphatase (ATPase) is expressed on the luminal membrane CPECs where it exports Na^+^ into the CSF space, creating an osmotic gradient permitting passive water export via AQP1 [53, 55]. AQP1 is absent on CPEC basal membrane and therefore does not mediate water permeability of the choroid plexus epithelial membrane as a whole. Interestingly, knockout of AQP1 in mice only causes a modest reduction in CSF secretion by about 20% [56], suggesting this is not vital for CSF production.

Another observation is that CSF secretion can occur against an osmotic gradient, incompatible with passive water movement via AQP1 as a primary mechanism of secretion [53]. This may be explained by the co-transport of water by luminal transporters including the Na^+^/K^+^/2 Cl^−^ cotransporter [NKCC1] and Na^+^/HCO3^−^ [NBCe2] co-transporter [57, 58]. Overall, accumulating evidence suggests that a variety of choroidal transporters are important in CSF secretion, coupling solute and water transport across the choroid plexus epithelium. There remain significant questions about the relative importance of individual transporters to this process.

### Physiology of CSF Clearance

Absorption of CSF occurs at several sites. Arachnoid granulations are protrusions of the arachnoid into the dural venous sinuses, providing outflow channels for CSF into the lumen of the venous sinuses [59]. Lymphatics provide another route of CSF absorption; drainage from the CSF space via cranial nerve sheaths and exit foramina, particularly the olfactory nerve, into the cervical lymphatic system has been recognised in rodents [60, 61].

More recently the glymphatic system has been described, using small fluorescent CSF tracers, confined by the BBB, to demonstrate movement from the CSF compartment into brain parenchyma [62, 63]. In this proposed system [64], fluid derived from CSF influx at the brain surface travels along periarterial spaces and enters the interstitial spaces of the brain. Transport between perivascular spaces and brain interstitium is mediated by astrocytic endfeet gaps and aquaporin- 4 [AQP4]. Fluid is cleared along perivenous spaces of large cortical draining veins. Drainage from this space may be via the subarachnoid space and venous pathway or the dural lymphatic system, the presence of which has now been demonstrated in human imaging studies [65, 66].

Using an intrathecally-administered contrast agent and MRI imaging to trace CSF drainage in humans, one study demonstrated drainage of CSF tracer to cervical lymph nodes, the timing of which was delayed compared to that seen in rodents [67]. Lymph node enhancement coincided with peak glymphatic enhancement suggesting a more important role for glymphatic-lymphatic connections in humans than the CSF-lymphatic connections (such as those around cranial nerve sheaths) seen in rodents [67]. Studying these pathways in vivo in humans is challenging, and the precise nature of connections between CSF spaces, brain, glymphatic and lymphatic systems is to be established [61].

### CSF Production and Clearance is Altered in IIH

CSF excess in IIH may be driven by either increased production of CSF, impaired clearance, or both. CSF dynamics are challenging to study in humans. Imaging studies utilising phase contrast cine MRI have demonstrated increased CSF flow in the cerebral aqueduct in IIH, suggesting increased CSF production at the choroid plexus, which reduces with treatment [68, 69]. Invasive measurement of ICP with intracranial monitoring or lumbar puncture can also provide information about CSF dynamics. ICP follows a pulsatile waveform related to the cardiac cycle, and fluctuates with changes in body position and potentially with circadian cycles [70, 71]. Studies utilising invasive ICP monitoring in IIH demonstrate the core feature of raised intracranial pressure alongside raised ICP pulse amplitude, indicative of reduced craniospinal compliance, and resistance to CSF outflow [72–74].

Animal models have been developed to study the relationship between obesity and CSF dynamics. Zucker rats have a leptin receptor mutation leading to significant obesity. An initial study found these rats had a higher ICP than lean controls but there was no significant rise in ICP with weight gain over a 28-day study period [75]. CPEC AQP1 was found to be more highly expressed in obesity but there were no changes in the Na^+^/K^+^ ATPase expression [75]. High-fat diet [HFD] has been used to induce obesity in rats. In one study, female rats fed HFD demonstrated increased CSF secretion compared with controls, but did not demonstrate increased resistance to CSF drainage [76]. These findings contrast with a later report in a similar model of HFD rats finding raised ICP and increased CSF outflow resistance without increased CSF secretion [77]. The authors postulated this discrepancy may be due to differences in the rate of weight gain, which was much greater in the first study, or related to technical factors such as the necessary use of anaesthesia in obtaining measurements [78]. In a further study, HFD rats demonstrated weight gain with an associated increase in ICP, increased retinal nerve fibre layer thickness and cephalic cutaneous allodynia indicating a broader IIH-relevant phenotype [79].

### Systemic Changes in IIH Drive CSF Hypersecretion

Many of the systemic metabolic changes seen in IIH have been linked to changes in CSF secretion.

#### Androgens

Human choroid plexus expresses androgen receptors, in addition to androgen activating enzymes [35]. Expression of membrane transporters in choroid plexus has been shown to vary in relation to the oestrus cycle, correlating with levels of androstenedione and progesterone, in rats [80]. In rat CPECs, testosterone was found to increase Na^+^/K^+^ ATPase activity which, as outlined above, has a role in CSF secretion [35]. Testosterone administration in lean female rats, mimicking the elevation in CSF testosterone seen in IIH, resulted in increased CSF production and raised ICP after four weeks of treatment [77]. Contrary to the result above, this was not associated with increased Na^+^/K^+^ ATPase activity but was associated with increased NKCC1 activity [77]. In another study, female obese Zucker rats were found to have no differences in ICP or CSF secretion compared with lean controls [81]. However, administration of testosterone resulted in increased CSF secretion rates [81]. Interestingly, this did not result in raised ICP and further analysis utilising CSF infusion studies found reduced CSF outflow resistance in the testosterone-treated rats [81]. Together this suggests testosterone drives hyperdynamic CSF circulation, with increased production and outflow.

#### Glucocorticoids

The choroid plexus also expresses 11βHSD1 [41, 82]. It has been proposed that increased 11βHSD1 activity in choroid plexus in IIH, with resultant increased glucocorticoid activity, may promote CSF secretion. Intraventricular hydrocortisone was shown to increase CSF secretion in female rats following either a high fat or control diet [76]. Against a prominent role for glucocorticoids driving the raised ICP phenotype in IIH is their directed use in other pathologies, such as tumours, to reduce ICP [83]. Whilst it may be that this is due to specific actions in the setting of pathological brain oedema, a recent study demonstrated that acute and chronic administration of prednisolone or corticosterone resulted in reduced ICP in adult female rats [84].

Treatment with an 11βHSD1 inhibitor [AZD4017] was shown in a phase II randomised trial to reduce ICP at 12 weeks, but this reduction was not significant compared to the placebo arm [85]. ICP reduction correlated with reduced serum cortisol:cortisone. Improved metabolic parameters including lipid profile and lean muscle mass have also been reported with 11βHSD1 inhibition [86]. Overall, there is evidence for a role of glucocorticoids and 11βHSD1 dysregulation in IIH, but whether the direct action of glucocorticoids on CSF secretion is a key driver of raised ICP is uncertain.

#### Cytokines

The effect of cytokines reported to be elevated in CSF in IIH on CSF dynamics has been explored in female rodents [76]. IL- 6, IL- 17, CCL2 and TNFα were administered intraventricularly to female rats following either a control or high-fat diet [76]. TNFα resulted in increased CSF secretion only in those fed the control diet, whilst the others did not significantly alter CSF secretion [76].

### Mechanisms of Impaired CSF Clearance in IIH

Increased CSF outflow resistance in IIH is likely to be multifactorial. Sites of interest where such an effect may be mediated in the CSF absorption and outflow pathways include the glymphatic and cranial lymphatic systems and cranial venous system.

#### Venous Outflow Resistance

A pressure gradient between the subarachnoid space and venous sinuses is important for CSF reabsorption, with pressure in the subarachnoid space required to be 3–5 mmHg greater than venous sinus pressure to facilitate this [87]. Intuitively, it has been suggested that increased intra-abdominal pressure in obesity transmits to raised central venous pressure, impeding venous return from the brain [88]. However, this does not account for the fact that most people living with obesity do not develop IIH, and this cannot be accounted for by differences in fat distribution which are now known to be similar in IIH and obesity alone [12, 89].

The role of venous sinus stenosis (VSS) in IIH is of increasing interest. An initial study reported VSS, evaluated by magnetic resonance venography (MRV), in 27/29 patients with IIH [90], whilst a recent MRV study found VSS in 60% of IIH patients [91]. In the latter study, presence of VSS was not associated with clinical outcomes including vision and headache [91]. Some consider that VSS in IIH is a secondary phenomenon due to extrinsic compression from raised ICP. This is supported by observations of reversal of VSS following CSF diversion [92, 93], and also recurrence of stenosis in stent-adjacent locations following VSS stenting [94]. Secondary VSS may lead to a positive feedback loop whereby resultant venous congestion contributes to reduced CSF absorption, further increasing ICP [87, 95]. Therefore, VSS may contribute to worsening of raised ICP in IIH but the initiating event in this cycle is likely to be raised ICP due to an alternative mechanism in the majority of patients with IIH [96].

#### Glymphatic Dysfunction in IIH

The description of the glymphatic system as an important mediator of brain water transport has led to hypotheses that dysfunction of this system may be important in the pathogenesis of IIH [97].

Imaging studies have provided some evidence that glymphatic dysfunction is present in IIH. Following intrathecal administration of the CSF tracer gadobutrol to patients with IIH, increased tracer enrichment with delayed clearance in various brain regions was found, suggesting impaired glymphatic function [98]. Diffusion tensor imaging has also been used as a surrogate marker of glymphatic function, demonstrating impaired diffusivity in perivascular spaces in IIH which correlated with grade of papilloedema [99].

Whilst these studies suggest impaired glymphatic function in IIH, they do not suggest its cause. Evidence suggests that the glia-neuro-vascular interface is disrupted in IIH, with reports of: changes in morphology and increased AQP4 expression at astrocytic endfeet [100]; astrogliosis [100, 101]; increased frequency of pathological mitochondria in astrocytic endfeet [102]; capillary damage and disruption of the BBB [101, 103]. These changes may reflect an inflammatory response, potentially due to BBB damage causing leakage of pro-inflammatory blood products or to systemic changes in IIH. Indeed, administration of the cytokine CCL2 to control and high-fat diet rats caused increased resistance to CSF drainage [76]. Evidence of dysfunction at the level of the glymphatic system and glial-neuro-vascular interface requires further exploration as to whether changes are permanent and whether they contribute to symptoms of IIH in particular, cognitive dysfunction [98].

## Insights from Cases that Fall into the Spectrum of IIH

The focus of this review has been on the mechanisms relevant to the “typical” IIH phenotype, with a striking association with obesity and female gender. Cases where the diagnostic criteria for IIH are fulfilled in patients without this phenotype may provide insights into pathological mechanisms by which raised ICP may occur.

### Transgender Patients with IIH

Multiple authors have reported cases of raised ICP in transgender patients, predominantly in those undergoing female-to-male gender-affirming hormone treatment with exogenous testosterone [104–114]. Most cases reported have also been classed as overweight, with BMI over 25 kg/m^2^, although a case with normal BMI has been reported [108]. These cases lend support for an important role of androgen excess in the development of raised ICP in IIH.

### Male Patients with IIH

Whilst a clear majority of IIH cases occur in females, around 13% of cases occur in males [116]. Several case series have reported on differences in clinical presentation, demographics and associated factors in males with IIH. Clinically, fewer men present with headache, but there appears to be an increased rates of visual disturbance and a higher risk of severe vision loss [117]. An initial report suggested a lower frequency of being ‘significantly overweight’ in males with IIH [118] but this was not defined and a larger series did not find a significant difference in BMI between male and female patients with IIH [117]. Obstructive sleep apnoea (OSA) is more frequent in males compared to females with IIH [117, 119]. Finally, a case–control study identified that men with IIH reported significantly more symptoms of hypoandrogenism compared to matched controls [120]. Cases of IIH in males have been reported in the setting of primary hypogonadism [121] and androgen deprivation therapy [122]. This contrasts with the hyperandrogenism associated with IIH in females, and it has been suggested that this could reflect a pathological ‘window’ of testosterone levels in IIH [20]. This sexually dimorphic association of androgens in IIH is consistent with the adverse metabolic phenotype is observed in males with hypoandrogenism and females with hyperandrogenism, more generally [123].

## Conclusions

The increasing evidence suggests that IIH is characterised by unique systemic metabolic aberrations with insulin resistance, hyperleptinaemia, hyperandrogenism, systemic and tissue-level corticosteroid dysregulation, metabolomic changes, a systemic pro-inflammatory state and increased risk of cardiovascular disease. These changes can lead to alterations in CSF production and clearance, predominantly CSF hypersecretion, and we suggest that this is the initiating event leading to raised ICP in IIH. Resistance to CSF outflow is also present in IIH and this is likely to be important in preventing hyperdynamic CSF circulation which could compensate for a hypersecretory state. In this perspective, venous sinus stenosis is likely a secondary event which leads to a positive feedback loop driving further increases in ICP.

Weight loss is presently the only disease-modifying therapy in IIH [124], reflecting the importance of obesity and adipose tissue, with its unique metabolic profile, to the underlying pathophysiology. As rapid weight gain appears to be a major risk factor for IIH, targeting the observed dysregulated androgen pathways may be a novel future treatment option, as it may be that androgen excess is part of the initial trigger for IIH and the factor propagating the disease sequalae by driving CSF hypersecretion. Clinicians should be aware that IIH has an adverse metabolic phenotype with important associations and risks outside of those related to raised ICP and papilloedema. Treatments which act primarily to reduce CSF secretion, such as acetazolamide, or increase CSF drainage, such as CSF diversion or venous sinus stenting, will not address these other important associations that confer morbidity. Improved understanding of the metabolic dysregulation in IIH and how this produces raised ICP is leading to exploration of novel targeted treatments.

## Key References

- Hornby C, Botfield H, O’Reilly MW, Westgate C, Mitchell J, Mollan SP, et al. Evaluating the Fat Distribution in Idiopathic Intracranial Hypertension Using Dual-Energy X-ray Absorptiometry Scanning. Neuro-Ophthalmology 2018;42:99–104. 10.1080/01658107.2017.1334218.
  - Study utilising DEXA imaging to accurately assess fat distribution in IIH, finding this is similar to simple obesity and predominantly truncal.
- Westgate CSJ, Botfield HF, Alimajstorovic Z, Yiangou A, Walsh M, Smith G, et al. Systemic and adipocyte transcriptional and metabolic dysregulation in idiopathic intracranial hypertension. JCI Insight 2021;6:e145346. 10.1172/jci.insight.145346.
  - Study comprehensively evaluating biopsied adipose tissue in IIH compared to matched controls, finding metabolic and transcriptional changes associated with lipogenesis indicating priming for weight gain.
- Adderley NJ, Subramanian A, Nirantharakumar K, Yiangou A, Gokhale KM, Mollan SP, et al. Association between Idiopathic Intracranial Hypertension and Risk of Cardiovascular Diseases in Women in the United Kingdom. JAMA Neurol 2019;76:1088–98. 10.1001/jamaneurol.2019.1812.
  - Large cohort study demonstrating increased risk of cardiovascular disease, hypertension and type 2 diabetes in IIH compared with age and BMI matched controls.
- Mollan SP, Mitchell JL, Ottridge RS, Aguiar M, Yiangou A, Alimajstorovic Z, et al. Effectiveness of Bariatric Surgery vs Community Weight Management Intervention for the Treatment of Idiopathic Intracranial Hypertension: A Randomized Clinical Trial. JAMA Neurol 2021;78:678–86. 10.1001/jamaneurol.2021.0659.
  - Clinical trial finding that weight management in IIH with bariatric surgery resulted in improved ICP and quality-of-life metrics, sustained at 12 and 24 months.
- O’Reilly MW, Westgate CSJ, Hornby C, Botfield H, Taylor AE, Markey K, et al. A unique androgen excess signature in idiopathic intracranial hypertension is linked to cerebrospinal fluid dynamics. JCI Insight 2019;4. 10.1172/jci.insight.125348.
  - Study identifying hyperandrogenism in IIH with differences in androgen profile compared with simple obesity and PCOS.
- Alimajstorovic Z, Pascual-Baixauli E, Hawkes CA, Sharrack B, Loughlin AJ, Romero IA, et al. Cerebrospinal fluid dynamics modulation by diet and cytokines in rats. Fluids Barriers CNS 2020;17. 10.1186/s12987-020-0168-z.
  - Rodent study finding increased CSF secretion in rats fed high-fat diet, and following TNF-α administration.
  - Rodent study finding testosterone administration to lean female rats resulted in raised CSF secretion and ICP.
- Wardman JH, Andreassen SN, Toft-Bertelsen TL, Jensen MN, Wilhjelm JE, Styrishave B et al. CSF hyperdynamics in rats mimicking the obesity and androgen excess characteristic of patients with idiopathic intracranial hypertension. Fluids Barriers CNS 2024;21. 10.1186/s12987-024-00511-1.
  - Rodent study finding that administration of testosterone in very obese rats resulted in increased CSF secretion and hyperdynamic CSF circulation without increased ICP.
- Eide PK, Pripp AH, Ringstad G, Valnes LM. Impaired glymphatic function in idiopathic intracranial hypertension. Brain Commun 2021;3:fcab043. 10.1093/braincomms/fcab043.
  - CSF tracer study identifying impaired CSF clearance in multiple brain regions in IIH indicating dysfunction of glymphatic pathways.

## Data Availability

No datasets were generated or analysed during the current study.
